# Nanocellulose in tissue engineering and bioremediation: mechanism of action

**DOI:** 10.1080/21655979.2022.2074739

**Published:** 2022-05-24

**Authors:** Sherin Jacob, Reshmy R, Sherly Antony, Aravind Madhavan, Raveendran Sindhu, Mukesh Kumar Awasthi, Mohammed Kuddus, Santhosh Pillai, Sunita Varjani, Ashok Pandey, Parameswaran Binod

**Affiliations:** a Department of Biochemistry, Pushpagiri Institute of Medical Sciences and Research Centre, Thiruvalla, India; b Department of Science and Humanities, Providence College of Engineering, Chengannur, India; c Department of Microbiology, Pushpagiri Institute of Medical Sciences and Research Centre, Thiruvalla, India; d Mycobacterium Research Laboratory, Pathogen Biology Division, Rajiv Gandhi Center for Biotechnology, Jagathy, Thiruvananthapuram, India; e Department of Food Technology, T K M Institute of Technology, Kollam, India; f College of Natural Resources and Environment, Northwest a & F University, Yangling, China; g Department of Biochemistry, College of Medicine, University of Hail, Hail, Saudi Arabia; h Department of Biotechnology and Food Science, Durban University of Technology, Durban, South Africa; i Gujarat Pollution Control Board, Paryavaran Bhavan, Gandhinagar, India; j Centre for Innovation and Translational Research, CSIR- Indian Institute for Toxicology Research (CSIR-IITR), Lucknow, India; k Centre for Energy and Environmental Sustainability, Lucknow, India; l Sustainability Cluster, School of Engineering, University of Petroleum and Energy Studies, Dehradun, India; m Microbial Processes and Technology Division, CSIR-National Institute for Interdisciplinary Science and Technology (CSIR-NIIST), Trivandrum, India

**Keywords:** Nanocellulose, functionalization, mechanism, tissue engineering, bioremediation

## Abstract

Nanocellulose are nano-sized components which are biodegradable, biocompatible and renewable. It offers mechanical strength and chemical stability in plants and bacteria. The environmental contamination is reduced by employing various bioremediation techniques which usesmicroorganisms like algae, bacteria and fungi as bio-adsorbents. The bio adsorbent property of nanocellulose contribute more for the bioremediation methods and the detailed study of its mechanism and application is essential which is discussed here. The mechanism happening between the contaminant and nanocellulose adsorbent should be explored in detail in order to develop effective new bioremediation strategies. Nanocellulose structural functionalization helps to modify the nanocellulose structure based on which it can be utilized for specific functions. Exploring the mechanisms that contribute to the implementation of nanocellulose in tissue engineering helps for further developments and advancement in the biomedical application of nanocellulose. Not much studies are available that elucidate and study the basic steps involved in the biomedical and environmental usage of nanocellulose. This review has focussed on the basic mechanisms involved in the use of nanocellulose in tissue engineering and bioremediation processes.

## Graphical abstract

**Figure:**
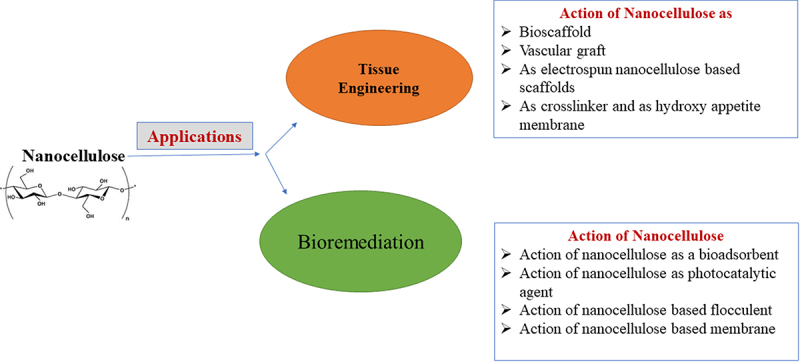

## Highlights

- Mechanism of nanocellulose in its various applications is not explored yet.
- Nanocellulose action in Tissue engineering via its functionalization is summarised.
- Nanocellulose action in bioremediation via its unique properties is enlisted.
- Limitations and future perspectives of nanocellulose and its application is analysed.

## 1. Introduction

Engineered nanomaterials and its applications in various aspects of nanotechnology have contributed to diverse requirements in biomedical fields as well as various environmental contamination prevention strategies [1]. Nanocellulose is formulated using 100 nano meter long tiny cellulose particles. The following are the specific attributes of a nanocellulose. It possesses high surface area. It is highly porous, has greater interconnection of pores, excellent stiffness, electrical conductivity, lower molecular mass, milder immunogenicity and extremely biodegradable [2]. Nanocellulose modification is significant as it creates alteration in characteristic features of it which can be explored for its various applications. Various surface modification or functionalization processes (oxidative reactions, carboxylation, ether addition, ester addition, silylation and grafting of polymer) helps to significantly improve the chemical properties of nanocellulose that is specifically required for its discrete applications [1–3]. The production and usage of nanocellulose have attained remarkable progress recently due to its increasing demand as a high-performance polymer. Cellulose molecule, which is the most abundant polymer, with 1–100 nanometer dimension is defined as nanocellulose. It is originated from native cellulose and exhibits amazing physical characteristics and very unique surface chemistry. It owes progressive demand in a wide variety of fields in biomedical engineering and bioremediation processes especially due to its anisotropic structure, significant biocompatibility, adaptive surface chemistry, renewability, amazing optical, physical and mechanical characteristics [3,4]. The possible steps involved in the nanocellulose action in various tissue engineering and bioremediation process are not explored much. The main aim of the current review is to find out the mechanism of action involved in various application of nanocellulose in tissue engineering and bioremediation process.

### 1.1. Nanocellulose structure and its remarkable features

Nanocellulose (C_6_H_10_O_5) n_) is a long chain homopolysaccharide with repeating units of C_12_H_22_O_11_ containing dextrose (D-glucose) units with Beta-1-4-glycosidic linkage [5]. The structure of cellulose is C_6_H_10_O_5)n_ and it is composed of continuous arrangement of glucose/glucopyranose molecules which ultimately leads to a chair conformation [6]. The – OH groups present in the structure are directed to the axis of the ring and it forms the backbone [7]. This type of arrangement in cellulose is maintained by the intramolecular H bonding. The formation of polymers and length of the nanocellulose are mainly determined by the cellulose differentiation [6–9].

Nanocellulose is defined as the substance procured from cellulose which is a major component of plants and a smaller quantity in animals and bacteria, and owes nano dimensions structurally [10]. Based on its creation and technique of production, nanocellulose is divided in to cellulose nano crystals (CNC), cellulose nanocrystals (CNCs), cellulose nanofibrils (CNFs), bacterial cellulose (BC) [11,12]. Cellulose nanocrystals (CNCs) are generated by hydrolytic cleavage of cellulosic particles using acids like concentrated H_2_S0_4_ (sulfuric acid). This kind of hydrolysis at specific unstructured regions of cellulose creates remarkable crystalline surfaces [13]. However, the techniques result in formation of a highly purified CNC in which a few sulfate groups are formed as impurities [12,13].

Cellulose nanofibrils are elongated fibers of very minute diameter in the nano scale range. Cellulose nano fibrils are developed by applying high pressure that cause crushing of cellulose in its pulp form as a result, powerfully twisted fibril networks are generated [14,15]. Cellulose nanofibrils have coexistence of formless and crystal regions together in their structure. It is very minute in length and diameter.

Cellulose nanofibril can be obtained by the following methods (1) mechanical methods that involve homogenization and crushing (2) chemical methods involving oxidation with TEMPO (2,2,6,6-Tetramethylpiperidinyloxy) and (3) by utilizing the advantages of both procedures mentioned above [14–16].

Bacterial or microbial cellulose (BC) is constructed from Bacteria and it is free of lignin and other molecules which acts as contaminant. It doesn’t involve polymerization or combining of minute units for its formation [17]. Hence while comparing bacterial cellulose with cellulose nanocrystals and cellulose nanofibrils, the latter involve polymerization of minute molecules from very smaller sizes [2]. The glucose residues formed inside the bacteria are utilized to create bacterial cellulose and the chains formed comes out of the small pores present in the cell wall [14]. Hence, the typical shape of bacterial cellulose is due to the combination of the glucose chain with that of the cell wall which ultimately leads to the formation of a network of cellulose nanofibrils structures [12,17,18].

The preparation process of nanocellulose involves a pre-treatment method in which the wood cellulose fibers are undergone pre-treatment steps in order to minimize the energy consumption and increase the degree of production of nanofibers [18]. The following processes are the common pre-treatment methods executed during the synthesis of nanocellulose; (i) Enzyme hydrolysis; cellobiohydrolases, endoglucanases, laccase etc., are specific enzymes involved in modifying the disordered structure of cellulose without disturbing the cellulose content [19,20] (ii) Alkaline-acid pretreatment; NaOH (sodium hydroxide) enhance area available in cellulose fibers and improve cellulose yield and hydrochloric acid which solubilizes the hemicelluloses are commonly used for pretreatment method [21–23]. (iii) ionic liquids containing 1-butyl-3-methylimidazolium chloride helps in carrying out the homogenization process in the presence of elevated pressure during the synthesis of NFC [24] (iv) Mechanical process; converts cellulose fiber in to nanocellulose forms by mechanical processes like grinding, ultrasonication, homogenization, cryo crushing etc. [25,26], (v) Chemical hydrolysis; controlled acid hydrolysis treatment cause the entry of H^+^ ions to the specific regions of cellulose and cause hydrolysis of glycosidic bond [27,28]. The properties of nanocellulose are diverse and act as a carrier for various specific materials. It can be used as an effective nano reinforcement due to its equal distribution property and powerful adhesive capacity [29,30]. The hydroxyl (-OH) group at C- 6 of cellulose structure can react with a speed of multitude times than the hydroxyl groups on the 2^nd^ position which was found to be twice at the 3^rd^ position [31]. Biocompatibility, hemocompatibility and biodegradability are one of the major important qualities of nanocellulose which helps to explore new possibilities of its applications in different areas [32].

### 1.2. Significance in tissue engineering and bioremediation

**Figure 1.**
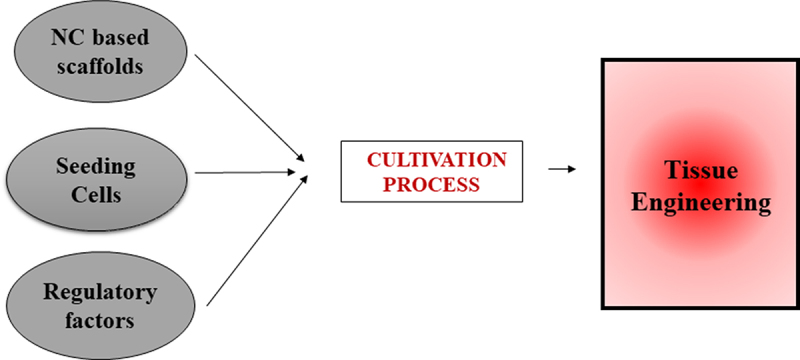
Schematic representation of basic factors involved in the cultivation process during tissue engineering.

Tissue engineering (TE) involves isolating and inoculating specific cells in scaffolds with the regulation of biochemical factors to construct functional substitutes. The application of TE involves repairing and regeneration of skin, blood vessels, muscles, intervertebral discs, bones, ligaments etc [33]. Seeding cells, regulatory factors and scaffolds are three essential elements of TE (Figure 1). Βeta-1,4-glucose in the molecular chain of Nanocellulose contains three active -OH groups. Nanocellulose easily form H-bond that attribute to its high mechanical strength, tailorable surface modification, good hydrophilicity and biocompatibility [34]. Exploring the mechanisms contributing the implementation of nanocellulose in tissue engineering contributes to further developments and advancement in the biomedical application of nanocellulose.

Bioremediation is a process in which the environmental pollutants like heavy metals, etc., are either removed or converted in to less toxic/ harmful components there by protecting human and animal population from its adverse health and social effects. It can be used to purify soil and water [35,36]. Various bioremediation techniques are explored by using the microorganisms like algae, bacteria and fungi as bio adsorbents. The bio adsorbent property of nanocellulose which is functionalized by chemical and structural modification contributes more for the bioremediation applications and mechanisms that has discussed here. Nanocellulose show sublime adsorption ability, increased mechanical strength, hydrophilic properties, renewable nature and biodegrading property because of which it is chosen as a propitious bio-adsorbent for removal of contamination [37]. Nanocellulose shows greater ability to adsorb and enhanced capacity to bind effectively in comparison to other corresponding macroscale materials that perform bio-adsorption [38]. In addition to that, high specificity in area of the nanocellulose and sufficient amount of -OH groups within the structure contributes to the potential modification essential for decontamination or pollution remediation process [38]. The mechanism happening between the contaminant and nanocellulose adsorbent should be explored in detail in order to develop effective new bioremediation strategies. Nanocellulose structural functionalization helps to modify the nanocellulose based on the action it is intended to do in its various application areas [39,40]. This review has focussed on the basic biochemical mechanisms involved in the application of nanocellulose in tissue engineering and bioremediation processes.

### 1.3. Mechanism of nanocellulose in tissue engineering

#### 1.3.1. As bio-scaffold in skin tissue engineering

Nanocellulose encourages binding and multiplication of cells by acting as a tissue bioscaffold. It exhibits the biocompatibility and mechanical features resembling natural tissue. Different tissue types are cultured on nanocellulose-based materials. Bacterial cellulose possesses low cytotoxicity and high porosity [41,42]. In spin-coating method, a very minute layer of film with nanocellulose adsorbed to it is prepared. The specific sizes as well as shape of the nanocellulose facilitate the cells to adopt more densely oriented nanocrystals surface. The electrospun nanofibers together with nanocellulose can be used as the supporting scaffold to culture human cells [42].

**Figure 2.**
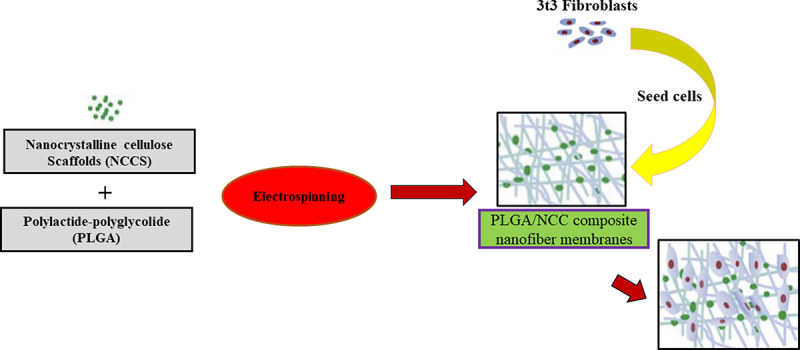
Schematic representation of nanocellulose based skin scaffolds. It represents the preparation of electrospun PLGA/NCC membranes with favorable biocompatibility.

Electrospinning causes stretching of polymer solution or it may lead to melting into small stream by the application of electric force followed by solidification, establishing continuous fibers from nano size to micro size range. By rearranging spinning characters, the electrospinning cause fibrous type of scaffolds which are tremendously porous in nature with sufficient surface area. It has been reported that the greater cell compatibility that showed adhesion and fibroblast proliferation and distribution was for the electrospun polylactide-polyglycolide (PLGA) nanocellulose membranes (Figure 2). It is reported previously that, the membrane loaded with 7 wt% of nanocellulose, showed features similar to native skin [41–45].

Freeze-drying (lyophilization) causes sublimation of the solvent to form the dry materials. The scaffolds maintain the initial structure and exact shape which results in wonderful quality after rehydrating [46]. Brown algae nanofibrillar cellulose (BANFC) suspension provided high porosity and water absorption to sponges. BANFC/quaternized chitin/organic rectorite (BANFC/QCR) sponges can be obtained by soaking in antibacterial suspension [47]. It provides mechanical strength and antibacterial property. It also accelerates skin healing by facilitating collagen formation and neovascularization [48]. The polyvinyl alcohol (PVA)/NCC scaffolds fabricated by freeze-drying showed interconnected pore structures, in which the pore size was found to be increasing a s the concentration of nanocrystalline cellulose was rising [49]. The mechanical, thermal and swelling features of scaffolds are prominently improved by the addition of nanocellulose. The uniform porous structures procured by freeze-drying and the hydrophilicity of nanocellulose provide a wonderful microenvironment for the cells to adhere, grow and metabolize effectively [22,23,49].

#### 1.3.2. As vascular graft in repairing of blood vessels

The strength and blood biocompatibility contribute to development of artificial tubes used for replacing minute vascular grafts. It possesses enormous water retention property, low roughness of inner tube surface. Nanocellulose is allowed to stick to the matrix formed with fibrin using covalent bonds and then it is provided with sufficient nano reinforcement [50]. Novel polyurethane reinforced with nanocellulose has significant application in vascular replacement due to its improved elastic properties along with excellent blood clotting property and good physical characters and mechanical features [50]. Electro spun scaffolds exhibit porous structures which are connected to each other and enhanced area which is indispensable for the cell growth and exchange of nutrients in vascular tissue engineering. Cellulose acetate and cellulose fibrous scaffolds incorporating nanocrystalline cellulose and microcrystalline cellulose are fabricated by electrospinning and deacetylation. Due to the presence of cellulose particulates, the scaffolds show multi-scaled structure, and will be similar to the natural extracellular matrix. Microcrystalline cellulose and Nanocrystalline cellulose acts synergistically in which former helps in anchoring of cells where as the later encourage cell adhesion. Enhancing the nanocrystalline cellulose and microcrystalline cellulose has substantially improved the viability and morphology [23,50].

The electron spun scaffolds possessing sustainable degradation can be developed by increasing poly (butylene succinate) (PBS) and poly (lactic acid) (PLA) with NFC. Nanofibrillar cellulose helps in betterment of fiber structure, tensile strength, elastic modulus and biocompatibility of scaffolds. Nanocellulose-based vascular scaffolds should have substantial strength to withstand the mechanical force developing in the blood vessels at different tissue sites [51]. Scaffolds with nanocrystalline cellulose and cellulose acetate propionate (CAP) are used for development of blood vessels with less diameter. Nanocrystalline cellulose possesses significant directional rigidity and strength to the scaffolds even at 0.2 wt%. The strong hydrogen bonding of nanocrystalline cellulose provided prominent strength outwardly and nanostructures with pores to the scaffolds, which in turn contribute to resistance to the physiological pressure and mimicking the features of the natural ECM in blood vessels [51]

Application of a small force of magnetic field makes a significant impact on the NCC – CAP solution. It makes NCC to get arranged perfectly in CAP matrix. The modification by application of magnetic field has enhanced the oozing and direction of nanoparticle [52]. It also declined the nanocrystalline cellulose concentration to get satisfactory properties. The minute vascular grafts which are of smaller diameter is functionalized by addition of mechanical characters, which in turn is altered based on the requirements of the tissue engineering needs [33,53].

#### 1.3.3. As electrospun nanocellulose based scaffolds in neural tissue engineering

Neuronal tissue cannot repair by itself and hence it is very difficult for neurons to regenerate after damage. Neural tissue engineering scaffolds should be able to direct the development of axon, modulate the axon channel and promote neural stimulation and activity in specific circumstances. Neurons utilize comparatively weak electrochemical signals to adjust cellular activities. The materials are capable of transmitting these signals and providing electrical stimulation for the growth of neurons [22]. Scaffolds with prominent performances specifically induce cell behavior which are significant for neural tissue engineering. The conductive materials are equipped with the ability to transmit these signals and provide electrical stimulation for the growth of neurons. Scaffolds with significant performances can correctly induce cell behavior, which possess greater significance to neural tissue engineering [23].

Electrospun nanocellulose-based scaffolds are suitable for the neural tissue engineering as it possess controllable number of pores, mechanical features, position and adaptability. The uniaxially aligned electrospun cellulose/nanocrystalline cellulose nanocomposite nanofibers [ECCNN] is constructed using a rotating drum which act as a collector of electrospin. By the addition of 20 wt% nanocrystalline cellulose, the elastic modulus and tensile strength of ECCNN in the direction of fiber orientation is increased and the thermal stability is also significantly enhanced. The cells might undergo significant proliferation rapidly not only on the surface of ECCNN but also deep inside. ECCNN- aligned nanofibers regulates cellular organization by providing scaffolds advantages on neural tissue engineering [53–56].

#### 1.3.4. As crosslinker and hydroxy apatite membrane in bone tissue engineering

Nanocellulose which are highly compatible for the tissues, highly durable and cause lesser levels of calcification are the choices to be considered for the replacement of cartilages. Bacterial cellulose/polyacrylamide gels are used as a crosslinker. These gels are able to furnish length and tensile strength similar to that of a ligament. Both of that showed properties attributed only to tendons, ligaments, etc. [57]. Nanocellulose is a promising scaffold for osteoblast and chondroblast. It can be used for the regeneration and repair of osteoblast cells of bone. Bacterial cellulose and hydroxyapatite containing membrane is potential for bone regeneration. It facilitates development and growth of bone cells, it causes enhanced ALP (alkaline phosphatase) enzyme activity, which is essential for the osteoblast functioning and also enhance the nodule growth and production in bone [37,58].

## 2. Mechanism of nanocellulose in bioremediation

**Figure 3.**
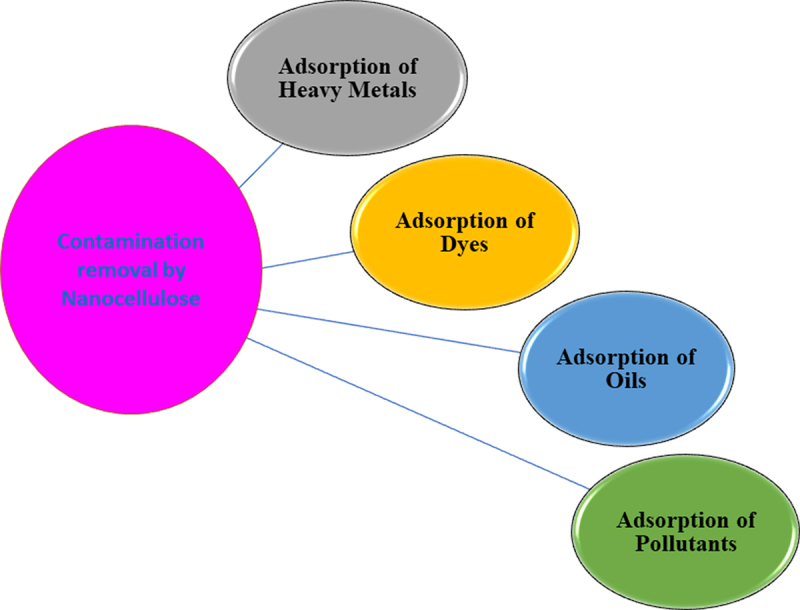
Schematic representation of pollutants removed in the contamination remediation by nanocellulose.

The native form of nanocellulose has several restrictions to be used as an adsorbent. Whereas, different functionalization processes can enhance the surface polarity and hydrophilic nature of nanocellulose. The adsorption of nanocellulose toward a particular contaminant depends upon the type of surface functionalization it has undergone (Figure 3). The functionalization of nanocellulose is modified based on the nature of the target contaminant [59].

The mechanism of action of adsorption process between the nanocellulose and the contaminant depends mainly on the type of surface modification in nanocellulose. If the pollutant is positively charged like heavy metals and dyes, nanocellulose possessing -ve charge facilitates the adsorption and removal of it. If the contaminant is oil which has hydrophobicity and oleophilic property, then the nanocellulose is modified by introducing hydrophobic groups that possess decreased surface energy and helps in adsorption to the nanocellulose. The following are the basic mechanisms involved in the surface functionalization and specific adsorbent capacity shown by the nanocellulose which is explored for the expulsion and removal of pollutants or contaminants [60].

### 2.1. Bioremediation using adsorption mechanism

Nanocellulose-based adsorbents are used for purification of waste water. Cellulose has to undergo certain modification process for it to perform the adsorption interaction on the pollutants. Nanocellulose are considered as effective natural adsorbent for wastewater purification because of its adsorption affinity to the organic contaminants. Advantage of using nanocellulose is that, the high surface area and high porosity prevent quantum size effects and also restrict the internal diffusion during bioremediation. Nanocellulose are used for the adsorption of residual antibiotics present in the industrial effluents, aquaculture system and in medical discharge wastes. The following are the various modifications that contribute to the bio adsorbent property of nanocellulose [32,43].

#### 2.1.1. (i) By In-situ TEMPO functionalization and amino functionalization

Carboxyl functionalized nanocellulose is formed by in-situ TEMPO functionalization. A support layer with two nanocellulose is developed in this modification. It helps to increase the permeability of water which is attributed to its porous network architecture. In this process, the nanocellulose functional layer is treated with TEMPO-Sodium Bromide, Sodium Hypochlorite system through in situ functionalization which is targeted to combine the -COOH (carboxyl) functional group. Carbonylated nanocellulose possess huge number of -COOH groups which is beneficial for metal-ion binding [61,62]. Various functionalization process leading to remediation of contamination by nanocellulose is listed in Table 1. Nanocellulose-based polyethyleneimine is formed by the process of amino functionalization of nanocellulose. The surface modification method of it includes cross-linking in which azidine combined with -COOH (carboxyl) group by a reaction that opens the ring structure at RT (room temperature) and has high efficacy. The established adsorbent exhibits a 3D multiple walled perforated cellular components with excess number of -NH2 (amino groups) and -O (oxygen) possessing groups. The amino groups attached will play a major part in the adsorption of copper related contaminants. The prepared adsorbent can easily anchor copper ions leading to the formation of tetradentate or bidentate coordination cyclic chelate [63,64].

**Table 1.** Functionalization of nanocellulose for bioremediation process

Sl. No	Functionalization	Contaminant Removed	References
1	In-Situ TEMPO Functionalization	Heavy metals	67, 68
2	Amino Functionalization	Heavy metals	68
3	Bridge effect	Heavy metals	69
4	Magnetic carboxylation	Heavy metals	70,73
5	Electrosteric stabilization	Dyes	71
6	Silanization	Oils	72
7	Stearoyl chloride functionalization	Oils	73

#### 2.1.2. (ii) By ‘bridge effect’, magnetic carboxylation and Thiourea functionalization

Nanocellulose are modified by adding polyethleneimine as well as glutaraldehyde along with the bridge effect shown by Fe^2+^ ions. In this process, carboxylation with TEMPO and crosslinkage with PEI through peptidic coupling, cause the modification of nanocellulose surface suitable for the requirement. The crosslinking between nanocellulose and PEI is made strong by the addition of glutaraldehyde. The TEMPO and PEI enhance the adsorption ability to transition metals. The Fe ions are responsible for the connection of polymers which are far apart from each other [63–65]. Carboxyl group attached nanocellulose offers a huge amount of -COOH group which helps in the metal ion binding. It is treated with Fe_3_O_4_ nanoparticles by co-precipitation method. This type of modification is involved in eliminating heavy metals from water. The carboxyl and hydroxyl groups formed at the adsorbent play a major role and they function as major site for the adsorption remediation process [65]. Thiourea-functionalized magnetic ZnO/nanocellulose is efficient in removing heavy metals as it contain magnetic Fe_3_O_4_ and ZnO. A facile chemical formulation is used for the preparation of thiourea-functionalized magnetic nanocellulose. The -OH, -NH2 and -S groups are responsible for the adsorption of heavy metals [66].

#### 2.1.3. (iii) By electrosteric stabilization and functionalization with stearoyl chloride

Electrosterically stabilized nanocellulose is produced by a two-step method. The functionalized particle possesses a bigger -ve charge, because of which it has bigger positive charge adsorption property [67]. Fabrication of adsorbent of nanocellulose and polyvinylamine showed effective removal of Congo red stain and reactive light-yellow dye with higher efficacy of adsorption [67, 76]. The nanocellulose is fabricated by treatment with stearoyl chloride for the effective removal of dyes. The stearoyl chloride possess good hydrophobic property and better leophilic properties. Hence, it has the ability to adsorb oils sufficiently. [77]

### 2.2. Bioremediation using photocatalytic degradation

Nanostructured cellulose-based hybrid photocatalysts can bring out photocatalytic degradation of various types of organic pollutants. Cellulose can show photocatalytic property under UV/ visible light irradiation. Photocatalytic waste water management can be done using cellulose-based metal oxide nanostructures which appears as thin film/membrane/ fibre under UV and visible light radiation. The derivatives of nanocellulose can effortlessly get adsorb on metal oxide surface layers and can generate -OH groups on the metal alkoxide surface. The TiO2, ZnO, Graphene oxide and Fe_2_0_3_ are the nanocellulose-metal oxide that are used as photocatalysts [40,50].

### 2.3. Bioremediation using nanocellulose based flocculent

**Figure 4.**
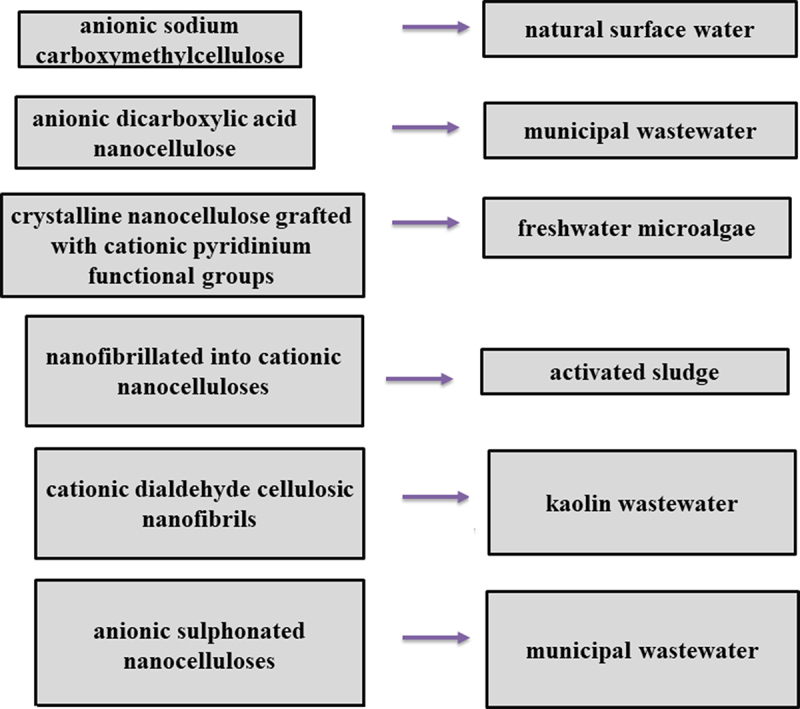
Application of cellulose-based flocculants in wastewater treatment.

Effective flocculants can be generated on the nanocellulose and the functionalization and is done by adding functional groups with anions, cations or hydrophobic groups on the nanocellulose surface via a neutralization process (Figure 4). This is achieved by the -OH group present on the cellulose surfaces which enables modification of nanocellulose to attach a preferable functionality on it and become a highly efficient flocculant [20,40].

## 3. Limitations and future perspective

The development of nanocellulose is a wonderful milestone as far as its application in tissue engineering and bioremediation is concerned. Several features of nanocellulose contributes its beneficiary effect in its fields of applications. However, the energy consumption during its production process and the mild toxicity reported from its end products are becoming a hindrance for the further exploration and application of this amazing compound in humans and in the environment. The future perspective of nanocellulose research should be focussed mainly on its minimal or nullified toxic manifestations in the human population. In the environmental remediation methods, the actual utility and all possible efficacy of nanocellulose in purifying the surroundings should be studied in detailed and should be able to implement those strategies on a large scale all over the world.

## 4. Conclusion

This review has focussed on the mechanism involved in the application of nanocellulose in tissue engineering and bioremediation process. We have analyzed the techniques employed by nanocellulose-based scaffolds in balancing mechanical features and porous architecture which are important for cell infiltration and proliferation. The review has analyzed the methods and specific functionalization processes applied in order to enhance the chemical characteristics of the surface of nanocellulose with respect to its application in the bioremediation process. However, while studying the significance and applications of nanocellulose for tissue engineering and bioremediation, we should be able to identify and rectify the adverse effects, if any, caused by this nanoparticle to human population. The mechanism of action has to be studied in detail by conducting specific experiments to know exact reactions and molecular changes that takes place on the nanocellulose surface and the corresponding components involved in both tissue engineering and bioremediation. Through this review, we have made a humble attempt to understand the mechanism behind the nanocellulose action in tissue engineering and bioremediation process, which will help us to expand new avenues of research and understanding to further utilize it for advanced applications.
